# Enhancement of the Visible Light Photodetection of Inorganic Photodiodes via Additional Quantum Dots Layers

**DOI:** 10.3390/mi15030318

**Published:** 2024-02-25

**Authors:** Seong Jae Kang, Jun Hyung Jeong, Jin Hyun Ma, Min Ho Park, Hyoun Ji Ha, Jung Min Yun, Yu Bin Kim, Seong Jun Kang

**Affiliations:** 1 Department of Advanced Materials Engineering for Information and Electronics, Kyung Hee University, Yongin 17104, Republic of Korea; andrew970728@khu.ac.kr (S.J.K.); 2016101120@khu.ac.kr (J.H.J.); majh0506@khu.ac.kr (J.H.M.); triolight@khu.ac.kr (M.H.P.); localha99@khu.ac.kr (H.J.H.); msd426@khu.ac.kr (J.M.Y.); bebeto@khu.ac.kr (Y.B.K.); 2 Integrated Education Program for Frontier Materials (BK21 Four), Kyung Hee University, Yongin 17104, Republic of Korea

**Keywords:** visible light photodetector, optoelectronics, quantum dots (QDs), band structure, metal oxide

## Abstract

Visible light photodetectors are extensively researched with transparent metal oxide holes/electron layers for various applications. Among the metal oxide transporting layers, nickel oxide (NiO) and zinc oxide (ZnO) are commonly adopted due to their wide band gap and high transparency. The objective of this study was to improve the visible light detection of NiO/ZnO photodiodes by introducing an additional quantum dot (QD) layer between the NiO and ZnO layers. Utilizing the unique property of QDs, we could select different sizes of QDs and responsive light wavelength ranges. The resulting red QDs utilized device that could detect light starting at 635 nm to UV (Ultra-violet) light wavelength and exhibited a photoresponsivity and external quantum efficiency (EQE) of 14.99 mA/W and 2.92% under 635 nm wavelength light illumination, respectively. Additionally, the green QDs, which utilized a device that could detect light starting at 520 nm, demonstrated photoresponsivity values of 8.34 mA/W and an EQE of 1.99% under 520 nm wavelength light illumination, respectively. In addition, we used X-ray photoelectron spectroscopy (XPS) and ultraviolet photoelectron spectroscopy (UPS) to investigate the origin of the photocurrents and the enhancement of the device’s performance. This study suggests that incorporating QDs with metal oxide semiconductors is an effective approach for detecting visible light wavelengths in transparent optoelectronic devices.

## 1. Introduction

Recent advancements in optoelectronic technology have led to an increasing demand for transparent photodetectors capable of detecting visible light [1,2,3]. Visible light detectors with high transparency and performance are essential for applications such as smart windows and glasses [4,5,6,7], medical diagnostic devices [8,9,10,11], security systems [12,13,14], and the autonomous vehicle industry [15,16]. The necessity for transparent, high-performance photodetectors across various fields primarily stems from their ability to process signals with precision and speed without visually intruding on or disrupting the aesthetics of their environment. Consequently, both the structural design of these devices and the materials used for composing their layers are subjects of extensive research aimed at improving the transparency and performance of photodetectors.

Highlighting the importance of high-performance photodetectors, it is essential to recognize the unique characteristics of three different types of photodetectors: photoconductors, photodiodes, and phototransistors, which are classified by their operating mechanisms [17,18]. Photoconductors are adept at low-light detection due to charge carrier recirculation but are slower in their responses. Photodiodes provide rapid responses and high efficiency, benefiting from a built-in potential, though they might necessitate complex circuitry. Phototransistors, offering signal amplification through a modulated channel, yield significant gain but can be complex and slower in their responses [19]. Given these factors, the choice of photodiodes in our research is further justified by their relative simplicity in fabrication, which allows for easier integration and scalability in various applications.

Additionally, metal oxide transporting layers are extensively studied in optoelectronic devices because of their high stability and transparency [20,21,22]. In this context, optimizing p-type metal oxide transporting layers is crucial in the optoelectronic field [23]. This significance stems from the relative scarcity of research on p-type metal oxide layers, particularly in contrast to the more extensive studies conducted on n-type metal oxide layers. By optimizing the properties of p-type layers, such as their electrical conductivity and optical transparency, we can significantly enhance the sensitivity and overall electrical performance of photodetectors. This approach not only addresses a gap in the current research but also opens new avenues for the development of advanced optoelectronic devices.

Furthermore, in terms of extending the detection range of a photodetector, various materials, such as perovskite [24,25], colloidal QDs [26,27,28], and organic materials [29,30] have been studied as active layers of photodiodes. Among these, colloidal QDs stand out due to their remarkable properties, which include tunable optical bandgaps with the size of the particles and high optical stability and quantum efficiency [31,32]. These properties imply that QDs are particularly suitable for enhancing the detection of visible light in photodetectors. Incorporating QDs into a photodetector specifically aims to enhance its sensitivity to a broader spectrum of visible light, addressing the limited wavelength detection capabilities of metal oxide layers. Both extending the detection range to visible light spectra and maintaining an overall response and efficiency can be achieved via the QD layer.

Here, we fabricated and optimized visible light detecting all inorganic photodiodes by utilizing an additional QD layer as an active layer via the spin-coating method in between the NiO and ZnO transporting layers. Firstly, we optimized the thickness of the NiO layer to enhance the electrical properties of the photodiode while maintaining transparent properties. In reverse bias, we successfully optimized the characteristics that suppressed the dark current while maintaining a highly transparent layer with approximately 85% transparency. To further understand the reasons behind this optimization, an XPS analysis was conducted. It revealed that changes in the oxygen vacancies in the NiO layer, depending on the thickness, contributed to performance enhancement. Subsequently, we utilized red QDs (RQDs) or green QDs (GQDs) between the NiO/ZnO structure. This approach allowed us to fabricate devices specifically responsive to 635 nm and 520 nm light by integrating different types of QDs between the NiO and ZnO layers, aiming to demonstrate a photoresponse within the visible light spectrum and assess the impact of incorporating different quantum dots into the structure. Both devices demonstrated fast response speeds and excellent photoresponsive characteristics. The visible light-detecting mechanism of these photodiodes was further elucidated through a UPS analysis and the band structure. These results demonstrated that inserting a QD layer in between the metal oxide transport layers could enhance visible light detection without degrading the performance of the photodiode, suggesting that the photodiode could be used in various applications with its high transparency and performance.

## 2. Experimental Section

### 2.1. Synthesis of the NiO Solution

A 1 M NiO solution was prepared by adding 2.9079 g of nickel(II) nitrate hexahydrate (Ni(NO_3_)_2_·6H_2_O, Sigma Aldrich, St. Louis, MO, USA) to 10 mL of N,N-Dimethylformamide (Sigma Aldrich, St. Louis, MO, USA). The solution was stirred at 60 °C for 1 h, followed by the addition of 100 μL acetylacetone (Sigma Aldrich, St. Louis, MO, USA) as a fuel of combustion method to produce the NiO layer with a comparably low temperature. Subsequently, the solution was stirred at room temperature for 24 h.

### 2.2. Photodiode Fabrication

Patterned indium tin oxide (ITO) glass substrates were cleansed via ultrasonication with deionized water, acetone, and isopropyl alcohol for 15 min in sequence. Following cleansing, the substrates were dried by blowing N_2_ gas. Subsequently, ultraviolet—ozone treatment was taken onto the surface of the ITO glass to remove the organic residues and improve the surface hydrophilicity of the substrates. For the fabrication of the photodiode, the NiO p-type layer was spin-coated onto an ITO glass substrate at 3000 rpm for 30 s. The spin-coated NiO layer was pre-annealed for 5 min at 120 °C to evaporate the residual solvent. Overall, 1~3 cycles of spin-coating and pre-annealing were undertaken. After the coating cycles, the NiO layer was post-annealed for 40 min at 250 °C. A solution of CdSe/ZnS RQDs or GQDs (UNIAM, Seoul, Republic of Korea, 20 mg/mL) dispersed in toluene was spin-coated onto the NiO layer at 2000 rpm for 30 s as an active layer of the photodiode, followed by annealing for 30 min at 180 °C. Then, the ZnO n-type layer was spin-coated with a 2.5 wt % ZnO solution (Avantama, Stafa, Switzerland, N-10) dispersed in isopropyl alcohol and annealed under the same conditions as the QD layer. All of the purchased materials were used without further processing. Finally, the Al electrodes (thickness: 130 nm) were deposited via thermal evaporation at a rate of 3 Å/s. The summarized fabrication process is shown in Table 1.

### 2.3. Characterization

Cross-sectional images and energy-dispersive spectroscopy (EDS) data were prepared to identify the structure of the device via HR-TEM (JEM-2100F, JEOL Ltd., Tokyo, Japan). The absorbance and transmittance of the films were measured using a UV–visible spectrometer (Cary 100, Agilent, Santa Clara, CA, USA). Both XPS and UPS spectra were measured in an ultra-high vacuum chamber. The XPS spectra were measured using K-Alpha (Thermo Electron, Waltham, MA, USA) with an Al-Kα (1486.6 eV) line source. The UPS spectra were measured using a NEXSA (ThermoFisher Scientific, Waltham, MA, USA) using a He-I (21.22 eV) line source with a −10 V bias applied on the sample during UPS measurement. The optoelectrical properties were measured using a probe station and a semiconductor parameter analyzer (HP 4145 B, Center for Detectors, Rochester, NY, USA). Various wavelengths (λ) of 635, 520, 450, and 405 nm were illuminated on a device with a laser diode with various intensities.

## 3. Results and Discussion

**Figure 1:**
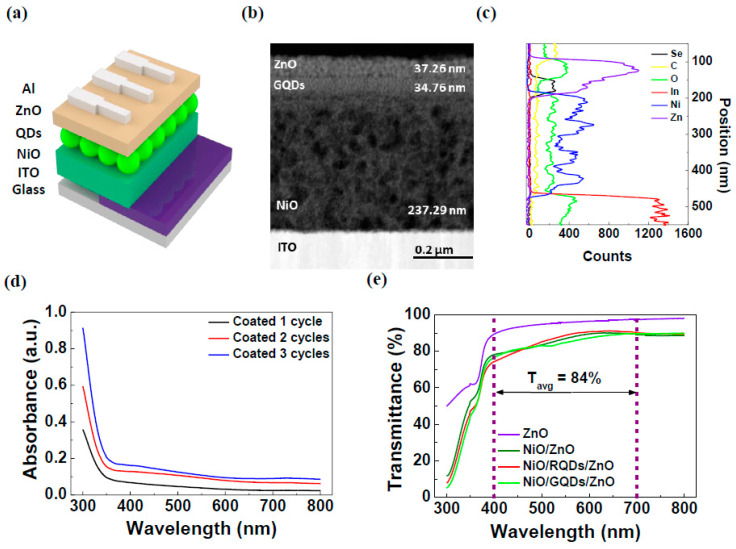
(**a**) Schematic of the fabricated photodiode. (**b**) Cross-sectional HR-TEM image of the fabricated photodiode. (**c**) EDS line scan of the photodiode. (**d**) Absorption spectra of the NiO layers. (**e**) Transmittance of the different layers.

Figure 1a presents a schematic diagram of the photodiodes fabricated in this study. The photodiodes were prepared by spin-coating NiO, QD, and ZnO layers onto an ITO/glass substrate, followed by the thermal evaporation of an Al metal cathode. The thicknesses of the individual layers were characterized using HR-TEM measurements, which revealed the optimized NiO layer had a thickness of 237.29 ± 2.37 nm after three cycles of coating. Similarly, the QD and ZnO layers exhibited thicknesses of 34.76 ± 0.35 nm and 37.26 ± 0.37 nm, respectively. The TEM and EDS data, presented in Figure 1b,c, confirmed that the stated thicknesses of the layers and each layer had no effect on the other layers. Additionally, the elemental mapping shown in Figure S1 further demonstrates that there was no effect between each layer. Figure 1d displays the absorbance spectra of the NiO layers with increasing NiO coating cycles. The results indicate that the absorbance spectra increased linearly with an increase in the number of coating cycles. Also, Figure 1d and Figure S2a–c showed that while NiO and ZnO absorb light in the UV region, RQDs and GQDs absorb light in the targeted visible spectrum. This result confirms that QDs are the primary contributors to the visible light photoresponse. In addition, Figure 1e shows the transmittance data of the devices, which decreased as the layers were stacked. The average transmittance (Tavg), calculated using the transmittance data in the 400–700 nm wavelength range, exhibited 86% for the NiO/RQDs/ZnO device and 84% for the NiO/GQDs/ZnO device. Based on the T_avg_ values obtained, it could be inferred that the NiO/QDs/ZnO photodiodes are transparent enough to hold potential for deployment as transparent visible light wavelength photodetectors with incorporation of transparent electrodes for various applications.

In addition, the performance of a photodiode, particularly related to dark current, is widely known to be influenced by the thickness of a device’s layers [33]. Specifically, the thickness of the transporting layer determines the width of the depletion region and affects the leakage current under reverse bias [34]. Therefore, it is crucial to optimize this aspect for improved device performance. Experiments adjusting the coating cycles in a NiO/QDs/ZnO structured device were conducted to optimize the performance related to leakage current, with the results presented in Figure S3. Figure S3a shows a decrease in the current under reverse bias with increased coating up to three times, indicating a reduced leakage current and dark current with thicker NiO layers. Furthermore, Figure S3b–d reveals the light response varying with the number of coatings, where the device with three cycles of NiO coating exhibited the best light response and lowest dark current, leading to the conclusion that it represents the optimized device configuration.

**Figure 2:**
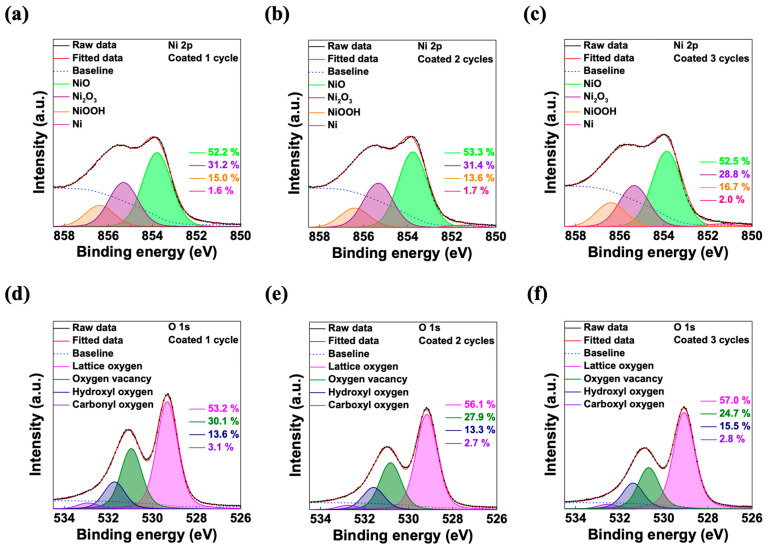
XPS spectra of NiO with different numbers of coating cycles. (**a**–**c**) Ni 2p_3/2_ and (**d**–**f**) O 1s core-level spectra.

To investigate whether the difference in the chemical components due to varying numbers of coating cycles affects the performance of the photodiodes, we conducted an XPS analysis. Figure 2 shows the Ni 2p_3/2_ and O 1s core-level XPS spectra of spin-coated NiO films with different numbers of coating cycles. The background was subtracted from the XPS spectra using a Tougaard-type background subtraction. Furthermore, the binding energies of the XPS spectra were calibrated, assigning the C 1s peak at 284.8 eV, and the peaks were standardized with the calibrated data. Figure 2a–c shows the Ni 2p_3/2_ peaks, which are primarily comprised of Ni^2+^ and Ni^3+^ peaks. The NiO peak at 853.82 ± 0.05 eV represents the Ni^2+^ peak, while the Ni_2_O_3_ peak at 855.34 ± 0.02 eV and the NiOOH peak at 856.40 ± 0.01 eV represents the Ni^3+^ peak [35]. Figure 2d–f shows the O 1s peaks of the NiO peaks, and they are convoluted with lattice oxygen (O_L_), oxygen vacancy (O_V_), hydroxyl oxygen (O_H_), and carboxyl oxygen (O_c_) peaks [35].

With p-type metal oxide, it is widely known that the composition of the metal ions and the amount of oxygen vacancies affect the hole mobility of thin films [36]. Since holes are the majority carriers of p-type metal oxide, reducing oxygen vacancies can enhance the hole concentration, potentially improving the conductivity. Figure S4a,b shows the area ratio of each peak in the Ni 2p_3/2_ and O 1s spectra. When changing the number of NiO coating cycles, the Ni^2+^/Ni^3+^ ratio was 1.15 ± 0.03 for every film, showing a similar composition of Ni ions. While the area ratios of the Ni peaks remained consistent across the different coating cycles, a notable trend was observed in the O 1s peaks. Specifically, the area ratio of the O_L_ peaks showed an increasing tendency, whereas the O_V_ peaks exhibited a decreasing tendency with an increase in the number of coating layers. As previously mentioned, the decrease in the oxygen vacancy leads to an increase in the hole concentration, which correlates with the improved performance of a photodiode. This improvement becomes evident with an increasing number of coating cycles of the NiO layer, suggesting an expected rise in the hole concentration within the NiO layer and the performance of the photodiodes.

**Figure 3:**
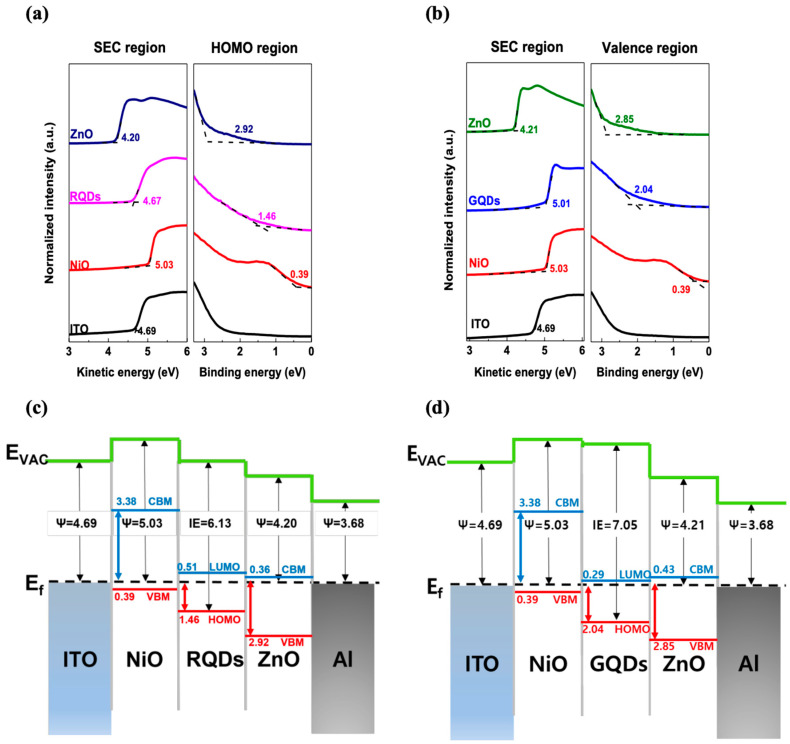
(**a**,**b**) UPS spectra of the different layers in the SEC region and valence region. (**c**,**d**) Energy level alignment and band structure of the photodiodes.

To figure out the band structure and light-detecting mechanism of the visible light-detecting photodiode, UPS measurements were taken. The configurations measured were ITO, ITO/NiO, ITO/NiO/RQDs, ITO/NiO/GQDs, ITO/NiO/RQDs/ZnO, and ITO/NiO/GQDs/ZnO. In Figure 3a,b, the work function of each layer was measured based on the kinetic energy of the electrons on the secondary electron cutoff (SEC) region. Furthermore, the energy difference between the fermi level (E_F_) and the valence band maximum (VBM) or the highest occupied molecular orbital (HOMO) is shown in the valence region. The work functions of each layer except the ITO were calculated as follows: 5.03 eV, 4.67 eV, 5.01 eV, 4.20 eV, and 4.21 eV. Moreover, the VBM (or HOMO) was calculated to be 0.39 eV, 1.46 eV, 2.04 eV, 2.92 eV, and 2.85 eV. In addition, the optical bandgap of each layer was calculated via a Tauc plot (Figure S5a–d). The calculated optical bandgap of the NiO, RQDs, GQDs, and ZnO layers was 3.77 eV, 1.98 eV, 2.33 eV, and 3.28 eV, respectively. Utilizing the optical bandgap values and UPS data, the conduction band minimum (CBM) or lowest unoccupied molecular orbital (LUMO) were therefore calculated to be 3.38 eV, 0.51 eV, 0.29 eV, and 0.43 eV, respectively [37]. Utilizing the calculated values, Figure 3c,d illustrates the comprehensive energy level alignment of the photodiode’s interfaces. The energy level diagram indicates that the RQDs and GQDs serve as the active layers for visible light detection in the photodiode. When visible light exceeds the respective bandgap illumination on the device, these quantum dot layers generate electron-hole pairs. Under reverse bias, these pairs are separated, contributing to the photocurrent generation within the device. The UPS measurements and the subsequent band structure analysis of the devices demonstrate that the incorporation of quantum dot layers between NiO and ZnO significantly enhances the capacity for visible light detection in the photodiodes with band alignment.

**Figure 4:**
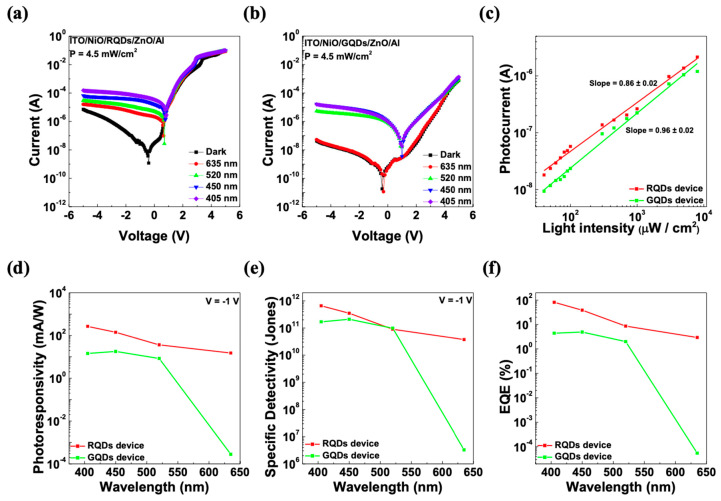
I-V curve characteristics of the (**a**) RQD device and (**b**) GQD device under various wavelength illumination conditions (635, 520, 450, and 405 nm). (**c**) Photocurrent—intensity curve for the fitting power law of the devices. (**d**) Photoresponsivity, (**e**) specific detectivity, and (**f**) EQE of each photodiode under various wavelength illumination conditions.

Figure 4a,b shows the current-voltage (I-V) characteristics of the photodiodes incorporating RQDs and GQDs. The curves demonstrate the devices’ photoresponse across a spectrum of incident light wavelengths as well as in the absence of light (the dark state). The power intensity (P) of the illuminated lights was ~4.5 mW/cm^2^. At −1 V bias, the photo-to-dark current ratio (*I_photo_*/*I_dark_*) of the RQD device under 635 nm light was 1.35 × 10^2,^ and the current ratio of the GQD device under 520 nm light was 1.67 × 10^3^. The *I_photo_*/*I_dark_* values of each device exhibited quite favorable numbers, which directly correlates with the signal-to-noise ratio. This implies that the device can detect light signals without being significantly affected by noise in dark conditions, ensuring reliable performance. In addition, the linearity of the photocurrent (*I_photo_*) versus the incident light intensity is important in evaluating the light-detecting performance of a photodetector. In Figure 4c, the linearity of the photocurrent on the light intensity of each device is shown according to the power law of *I_photo_*~P^α^. With simple power law fitting, α can be shown as the slope of the graph, and the value was 0.86 ± 0.02 for the RQD device and 0.96 ± 0.02 for the GQD device. The parameter α indicates the extent of electron-hole recombination or carrier scattering within the device; typically, values closer to 1 suggest better device performance. The devices we created also demonstrate performance approaching this optimal value, signifying their high efficiency. In Figure 4d–f, the photodetecting performance of the devices at −1 V bias was characterized in terms of photoresponsivity (R), specific detectivity (D*) [34], and external quantum efficiency (EQE), and each term was calculated using Equations (1)–(3) [34,38].

(1) $$R=\frac{I_{photo}-I_{dark}}{PS}$$

(2) $$D*=\frac{R}{\sqrt{(2qI_{dark})⁄S}}$$

(3) $$\mathrm{EQE}=\frac{Rh\nu }{e}\times 100\;(\%)$$

where *I_photo_* is the photocurrent, *I_dark_* is the dark current at −1 V, *P* is the intensity of the incident light, *S* is the area of the photodiode, *ν* is the frequency of the incident light, and *e* is the electron charge. The RQD device under 635 nm light exhibited photoresponsivity, specific detectivity, and an EQE of 14.98 mA/W, 3.73 × 10^10^ Jones, and 2.92%, respectively. The GQD device under 520 nm light exhibited 8.34 mA/W, 9.84 × 10^10^ Jones, and 1.99%, respectively. The performance of each device varied as the dark current of the devices were different; however, both photodiodes with QDs showed moderate performance on the desired wavelength of the incident light. The organized optical and electrical characteristics of the fabricated and similarly structured devices are shown in Table 2. While the values were slightly lower than those in the most recent references, including the advantage of easy fabrication, in conditions where QDs optimization was not directly undertaken, it demonstrated moderate values.

**Figure 5:**
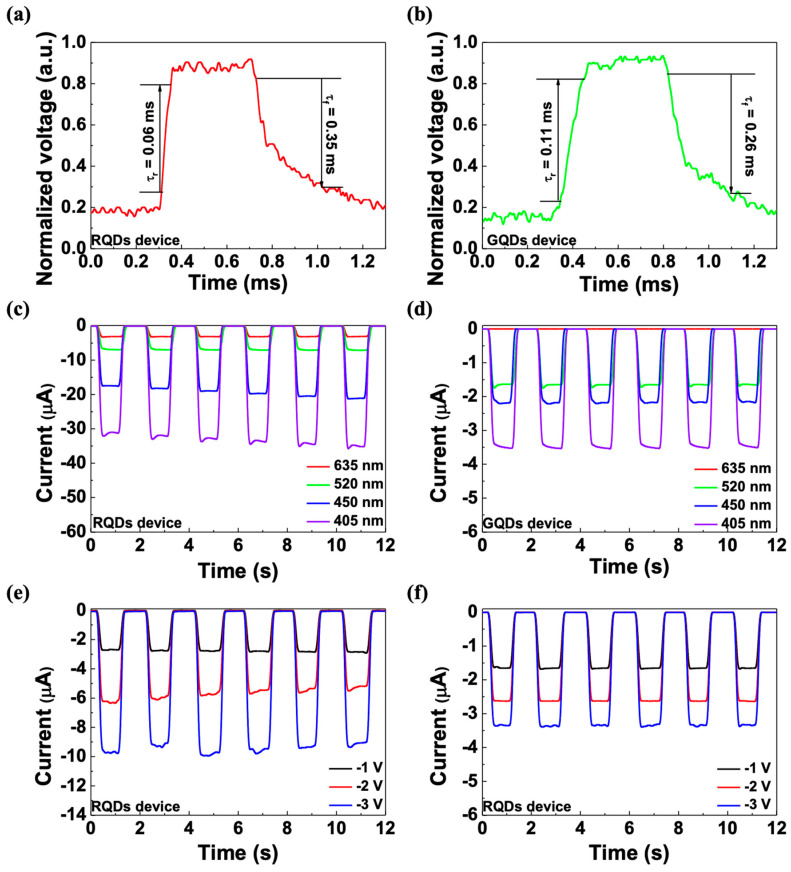
Rise/fall time of the (**a**) RQD device and (**b**) GQD device. I-T curve of the (**c**) RQD device and (**d**) GQD device under various wavelength illumination conditions at −1 V bias. I-T curve of the (**e**) RQD device and (**f**) GQD device at −1, −2, and −3 V bias under 635 nm and 520 nm light, respectively.

Figure 5a,b shows the rise (τ_r_) and fall (τ_f_) time of each device, which is calculated with the time between 10% and 90% of the maximum photocurrent. The rise and fall time of the device with RQDs was 0.06 ms and 0.35 ms, and the time of the device with GQDs was 0.11 ms and 0.26 ms, respectively. Both devices exhibited rapid rise and fall times, implying that the devices could be applied to applications that require fast responses. In addition, Figure 5c,f shows the photoresponses under different conditions. Each device shows constant responses under various wavelengths and different voltage biases. The results also imply that the device could be applied to circuits and used well because of their constant responses under various conditions.

## 4. Conclusions

In this paper, we fabricated all the inorganic photodiodes with an additional QD layer in between the NiO and ZnO layers to enhance the detectivity of visible light. The electrical and optical characteristics of the NiO layer were investigated through various analyses. To be specific, the I-V curve of the dark state and XPS measurement confirmed that three cycles of coating NiO film lead to the optimized thickness of the NiO layer. Furthermore, we showed the mechanism of enhanced visible light detection with a UPS measurement and band structure. Additionally, the photoresponse performance of each photodiode was shown with a photoresponsivity of 14.98 and 8.34 mA/W, a specific detectivity of 3.73 × 10^10^ and 9.84 × 10^10^ Jones, and an EQE of 2.92 and 1.99%, respectively. Lastly, the rise and fall time of the photodiodes were fast enough to be applied to various applications in the future. This study demonstrates that inserting an additional QD layer between the metal oxide p-n junction diode leads to a high-performance visible light photodetector.

## Figures and Tables

**Table 1:** Fabrication process of the photodiodes.

Procedure	Materials	Condition
Substrate cleaning	DI-water, acetone, IPA	Sonicating for 15 min per step
UV-ozone treatment	O_3_	Approximately 20 min
NiO spin-coating and thermal annealing	NiO solution	A coating rate of 3000 rpm (30 s) → 5 min annealing at 120 °C → 1~3 coating cycles → 40 min annealing at 180 °C
QDs spin-coating and thermal annealing	CdSe/ZnS (RQDs or GQDs)	A coating rate of 2000 rpm (30 s) → 30 min annealing at 180 °C
ZnO spin-coating and thermal annealing	ZnO nanoparticles (N10)	A coating rate of 2000 rpm (30 s) → 30 min annealing at 180 °C
Electrode deposition via thermal evaporation	Al	At a rate of 3 Å/s

**Table 2:** Summarization of the performance of the fabricated photodiodes and the similarly structured photodiodes.

Device Structure	Wavelength	*I_photo_*/*I_dark_*	Photoresponsivity (mA/W)	Detectivity (Jones)	EQE (%)	Rise/Fall Time (ms)	Ref.
ITO/NiO/RQDs/ZnO/Al	635 nm	1.35 × 10^2^	14.98	3.73 × 10^10^	2.92	0.06/0.35	This work
ITO/NiO/GQDs/ZnO/Al	520 nm	1.67 × 10^3^	8.34	9.84 × 10^10^	1.99	0.11/0.26	This work
ITO/Cu_2_SnS_3_–Ga_2_O_3_/CdZnSeS—ZnS QD/LZO/Al	628 nm	-	42.9	1.66 × 10^12^	8.46	2.1/2.6	[39]
ITO/Cu_2_SnS_3_–Ga_2_O_3_/CdZnSeS—ZnS QD/LZO/Al	540 nm	-	185.4	7.19 × 10^12^	42.57	-	[39]
ITO/CuO_x_/Pbs QDs/ZnO/Al	1010 nm		-	4.6 × 10^12^	15	0.009/0.010	[40]

## Data Availability

Data are contained within the article.

## Supplementary Materials

The following supporting information can be downloaded at: https://www.mdpi.com/article/10.3390/mi15030318/s1, Figure S1: Elemental mapping of representing elements of each layer utilized in fabricated photodiode. Figure S2: Absorption spectra of (a) RQDs, (b) GQDs, and (c) ZnO. Figure S3: I-V curves of (a) photodiodes with different number of coating cycles. (b–d) light response of photodiodes under visible light illumination. Figure S4: Area ratio of (a) Ni 2p_3/2_ spectra and (b) O 1s spectra. Figure S5: Tauc plot and optical bandgap of (a) NiO, (b) RQDs, (c) GQDs, and (d) ZnO layers.
