# Supplementary material for: Enhancement of the Visible Light Photodetection of Inorganic Photodiodes via Additional Quantum Dots Layers
Source: Micromachines (Basel). 2024 Feb 25;15(3):318. doi: 10.3390/mi15030318 (PMC10971844; doi:10.3390/mi15030318)
Supplement: Supplementary file 1 [file micromachines-15-00318-s001.zip › micromachines-2875998-supplementary.pdf]

Supplementary Information for:

Enhancement of Visible Light Photodetection of Inorganic Photodiodes via additional Quantum Dots Layer

Seong Jae Kang[1,2], Jun Hyung Jeong[1,2], Jin Hyun Ma[1,2], Min Ho Park[1,2], Hyoun Ji Ha[1,2], Jung Min Yun[1], Yu Bin Kim[1] and Seong Jun Kang[1,2,*]

[1] Department of Advanced Materials Engineering for Information and Electronics, Kyung Hee University, Yongin 17104, Republic of Korea

[2] Integrated Education Program for Frontier Materials (BK21 Four), Kyung Hee University, Yongin 17104, Republic of Korea

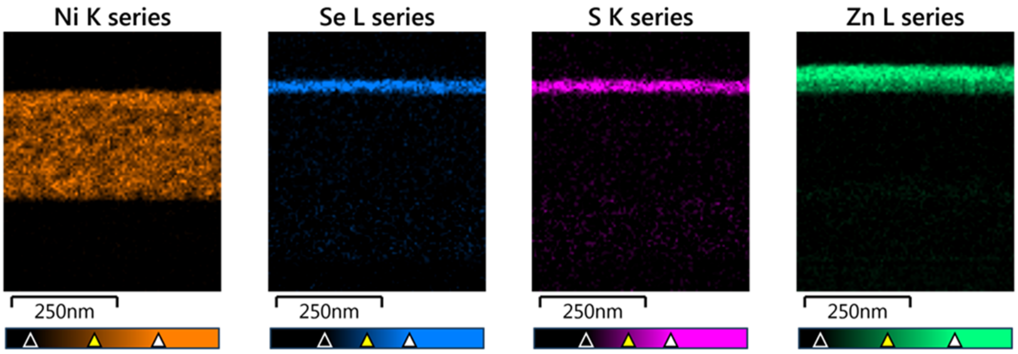

Figure S1. Elemental mapping of representing elements of each layer utilized in fabricated photodiode.

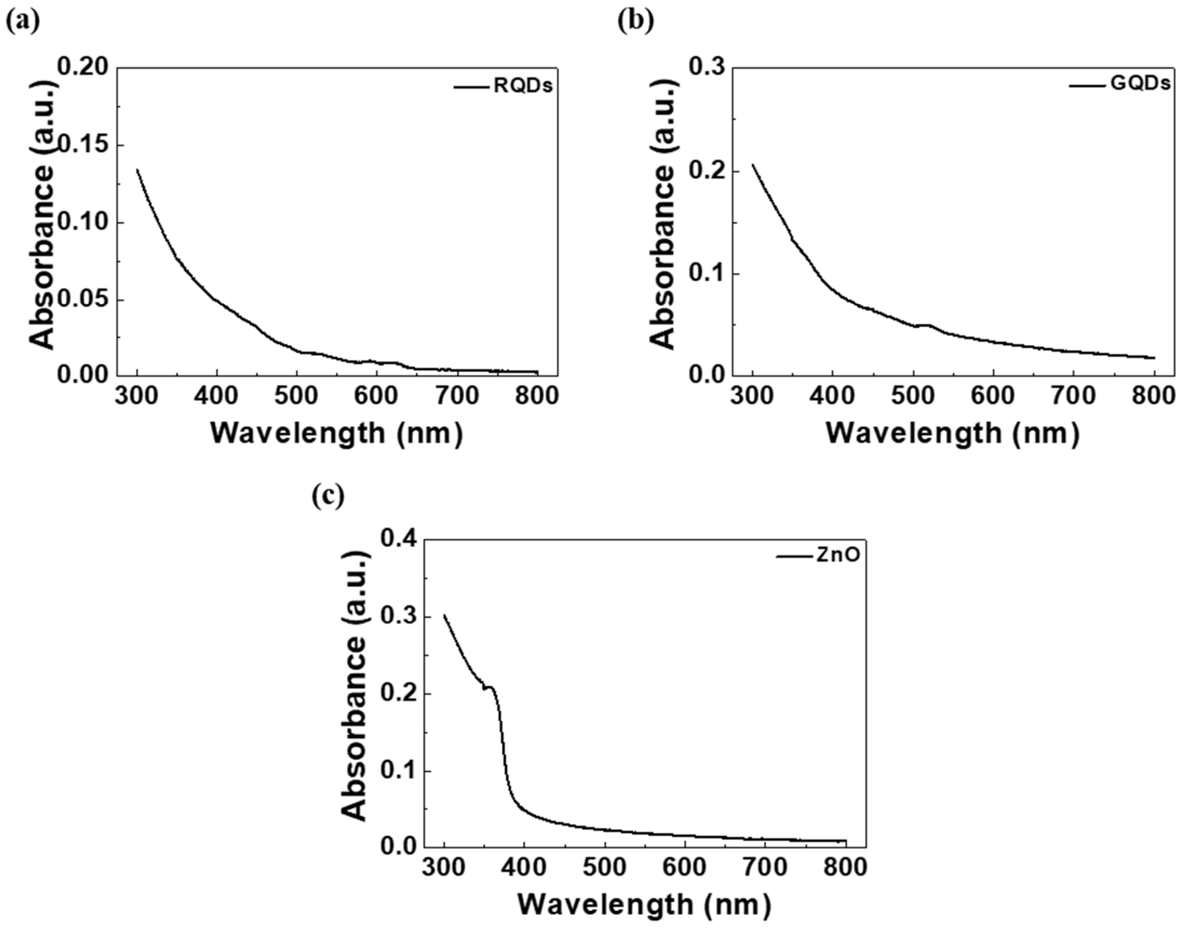

Figure S2. Absorption spectra of (a) RQDs, (b) GQDs, and (c) ZnO.

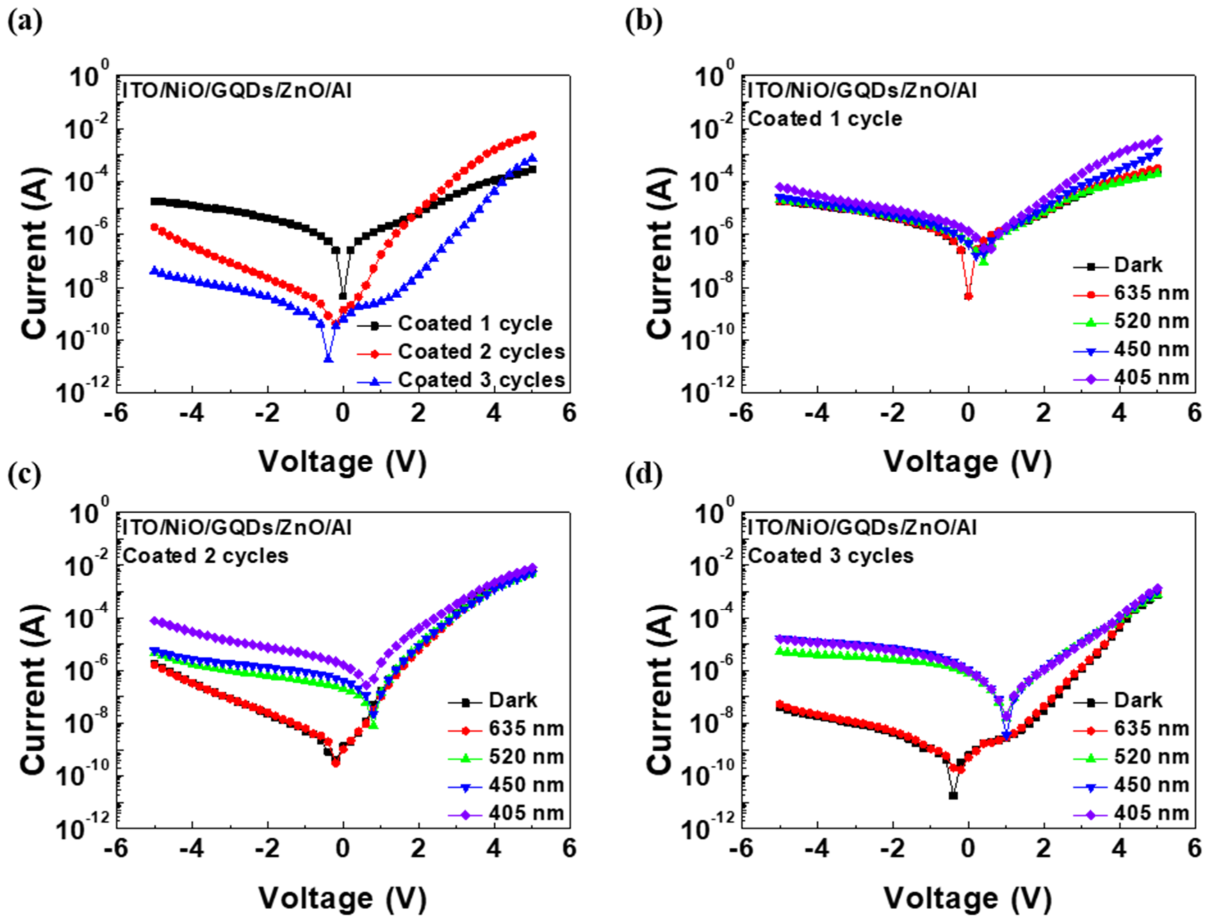

Figure S3. I-V curves of (a) photodiodes with different number of coating cycles. (b) ~ (d) light response of photodiodes under visible light illumination.

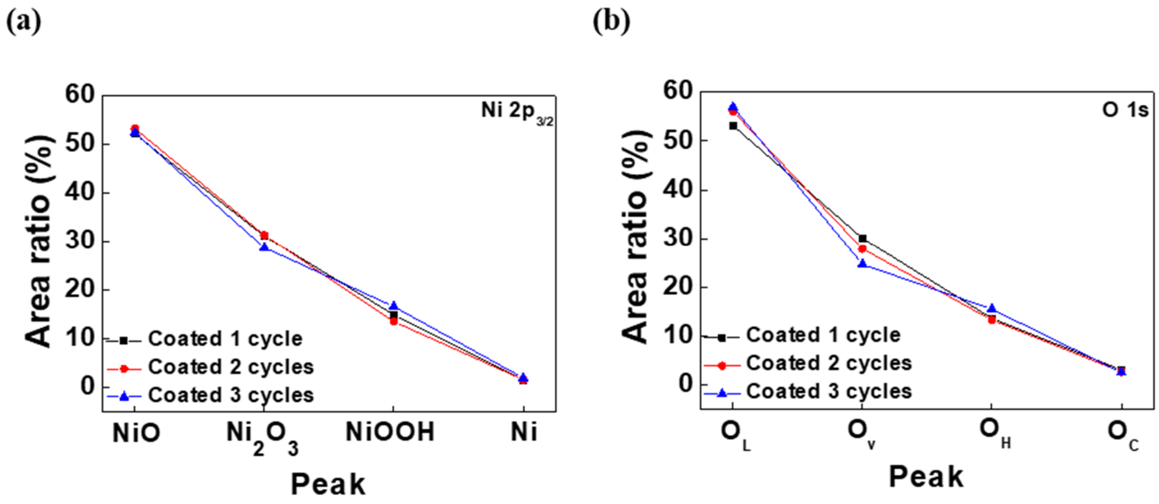

Figure S4. Area ratio of (a) Ni $2p_{3/2}$ spectra and (b) O 1s spectra.

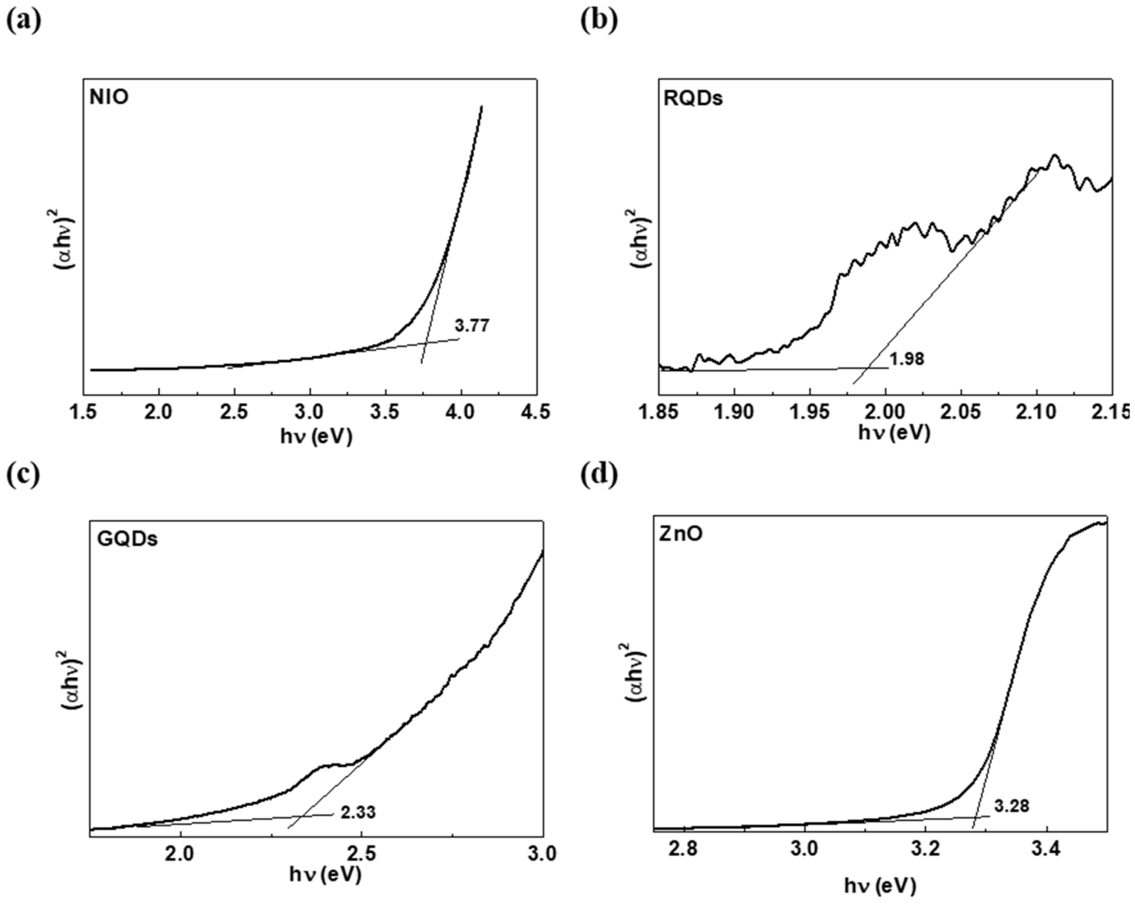

Figure S5. Tauc plot and optical bandgap of (a) NiO, (b) RQDs, (c) GQDs, and (d) ZnO layers.
